# Draft Genome Sequence of the Moderately Halophilic Gammaproteobacterium *Halomonas anticariensis* FP35^T^

**DOI:** 10.1128/genomeA.00497-13

**Published:** 2013-07-18

**Authors:** Ali Tahrioui, Emilia Quesada, Inmaculada Llamas

**Affiliations:** Department of Microbiology, Faculty of Pharmacy, University of Granada, Granada, Spaina; Biotechnology Research Institute, Polígono Universitarion de Fuentenueva, University of Granada, Granada, Spainb

## Abstract

*Halomonas anticariensis* strain FP35^T^ is a moderately halophilic bacterium isolated from a soil sample taken from Fuente de Piedra, a saline wetland in the province of Málaga (Spain), which produces an exopolysaccharide and quorum-sensing signaling molecules of the type *N*-acylhomoserine lactone. We report here the draft genome sequence of this gammaproteobacterium.

## GENOME ANNOUNCEMENT

*Halomonas anticariensis* FP35^T^ was isolated from saline soil at Fuente de Piedra, Málaga, in southern Spain (1). It is a Gram-negative, heterotrophic, aerobic rod and is motile by peritrichous flagella. This moderately halophilic organism has a respiratory metabolism, being able to grow in media with 0.5% to 15% (wt/vol) NaCl (optimal growth at 7.5% [wt/vol] NaCl). It excretes significant quantities of an exopolysaccharide (EPS) at the early stationary growth phase. This EPS is composed mainly of glucose, mannose, and galacturonic acid and produces solutions of low viscosity and pseudoplastic behavior. Moreover, it also has a high capacity for binding cations and has incorporated considerable quantities of sulfates, which is highly unusual in bacterial polysaccharides (2). This bacterium has also been reported to produce *N*-acylhomoserine lactones, which are signal molecules in quorum-sensing (QS) systems (3). We have characterized this system at the genetic level and found that it is composed of the *luxR* and *luxI* homologues *hanR*, believed to be the transcriptional regulator gene, and *hanI*, the autoinducer synthase gene (4). Furthermore, we have demonstrated that the *H. anticariensis* FP35^T^ QS system is regulated by a two-component system, suggesting its integral involvement in the intercellular communication strategies of this moderately halophilic bacterium (5). In addition, we recently demonstrated that the QS system is widespread within the *Halomonadaceae* family (6). *H. anticariensis* FP35^T^ belongs to the class *Gammaproteobacteria* within the family *Halomonadaceae* (7). *Halomonas* is one of the genera most frequently isolated from hypersaline habitats by conventional-culture techniques (8). Nevertheless, the ecological roles that *Halomonas* species play in these habitats and their relationships with other halophilic and nonhalophilic microorganisms are still unknown.

Little genome data are currently available from this group of bacteria. In this study, we report the draft genome sequence of *H. anticariensis* strain FP35^T^, which was obtained using high-throughput Illumina HiSeq paired-end (PE) sequencing technology. The raw data obtained were subjected to treatment, including several steps of data filtering. Then, we performed *de novo* assembly using SOAP*denovo* version 1.05 (9), generating 45 contigs that were further joined into 21 scaffolds. The assembled draft genome comprises 5,067,645 bases at 34.4-fold coverage and has a G+C content of 58.54%. The NCBI Prokaryotic Genomes Automatic Annotation Pipeline (PGAAP) (www.ncbi.nlm.nih.gov/genomes/static/Pipeline.html) was employed for gene annotation. The tRNA and rRNA genes were predicted with tRNAscan-SE (10) and RNAmmer (11), respectively. A total of 4,652 putative protein-coding genes (CDSs) or open reading frames (ORFs) were predicted. Furthermore, the strain FP35^T^ contains a total of 61 tRNA genes and one rRNA operon.

The draft genome information reported in the present study might be valuable for future studies to investigate the role of the quorum-sensing regulatory system in the adaptation to high salinities in hypersaline environments.

### Nucleotide sequence accession numbers.

The *H. anticariensis* strain FP35^T^ whole-genome shotgun project has been deposited at DDBJ/EMBL/GenBank under the accession no. ASTJ00000000. The version described in this paper is the first version, accession no. ASTJ01000000.
